# Pesticidal plant extract effect against major lepidopteran insect pests and their natural enemies in rice *Oryza sativa* L

**DOI:** 10.3389/finsc.2024.1500542

**Published:** 2025-01-07

**Authors:** Atanu Seni, Rini Pal, Sanjukta Mohapatra, Dipankar Mandal, Sushil Kumar Bansude, Pinki Seth, Sarita Barla, Jubuli Sahu

**Affiliations:** Odisha University of Agriculture and Technology, Regional Research and Technology Transfer Station, Sambalpur, Odisha, India

**Keywords:** botanicals, economics, insecticide, IPM, parasitoids, predators, yield

## Abstract

Extracts of plants have been used to manage various insect pests, but little information is available about how effective they are in reducing crop damage or how they affect crop yield and beneficial insects in rice. Extracts from *Azadirachta indica* leaves, *Lantana camara* leaves, *Nerium oleander* leaves, *Aegle marmelos* leaves, *Allium sativum* cloves, and *Citrus limon* fruits, known to have insecticidal properties, were compared with two checks, viz., Azadirachtin 1% EC and standard insecticide Acephate 95 SG, for their efficacy against yellow stem borer (YSB), *Scirpophaga incertulas* (Walk.), and rice leaf folder *Cnaphalocrocis medinalis* (Guenee) and natural enemies in cultivated rice in Sambalpur, Odisha, India. Untreated rice plants served as control. An adjuvant, Tween 20 at 1%, was added with all the botanical extracts except the commercial formulation. Plant damage, insect population numbers, and yield were monitored during two consecutive wet seasons from 2022 to 2023. Mean rice yield was significantly higher in the *A. indica* and Acephate 95 SG treatments, i.e., 4.68 t/ha and 4.66 t/ha, respectively, compared to the control (2.27 t/ha) and were significantly at par with each other. The *L. camara* and *A. indica* treatments were effective against both the major lepidopteran rice insect pests. The highest cost–benefit ratio of (1:4.65) was obtained from the Acephate treatment and was closely followed by the *A. indica* treatment (1:3.74). All the studied botanicals had less impact on natural enemies than synthetic chemicals. Among these botanicals, the maximum mean population of predators (like spiders and carabid beetles) and parasitoids (like *Tetrastichus schoenobii*, *Telenomus dignus*, and *Trichogramma japonicum*) were observed in the *A. indica* and *A. marmelos* treatments. Although all the studied botanicals were effective against both the major insect pests in rice, the *A. indica*, *A. marmelos*, *A. sativum*, and *L. camara* treatments showed the most promising against rice insect pests, so they may be incorporated into integrated pest management of rice.

## Introduction

Rice (*Oryza sativa* L.) belongs to the family Poaceae and is the staple food for one-third of the world’s population. In addition, it takes up nearly one-fifth of the total area of land used for cereals. It is cultivated in a wide range of geographical and cultural environments. Most of the world’s rice is cultivated and consumed in Asia, which constitutes more than half of the global population. Every year, rice is cultivated on approximately 11% of the world’s arable land. Next to China, India is the world’s second-largest producer and consumer of rice. Rice occupies a unique position in the Indian economy. In India, rice was grown on 46.38 million hectares in 2021–2022, with an annual production of 130.29 million tonnes and a productivity of approximately 2.81 t/ha (1). In Odisha, rice occupies an area of 3.94 million hectares with a production of 9.14 million tonnes and a productivity of 2.32 t/ha. It is the staple food of almost the entire population of Odisha, which accounts for 91% of the area under cereals and contributes approximately 94% of total cereal production in the state (Anonymous 2023). Introduction and wide cultivation of high-yielding varieties have led to the severe incidence of different insect pests. More than 15 species of various lepidopteran insect pests have been reported to attack the various stages of the growth period, out of which almost seven have caused notable damage (2–4). Among them, yellow stem borer (YSB), *Scirpophaga incertulas* (Walk.), and rice leaf folder *Cnaphalocrocis medinalis* (Guenee) share the prime importance to rice crop year after year. During the vegetative stage, the YSB larvae bore into the stem, severing nutrient flow and resulting in “dead hearts” where the central shoot turns yellow, wilts, and can be easily pulled out, often revealing the larva inside. In the reproductive stage, their feeding damage causes “whiteheads”, or panicles that turn white, remain upright, and fail to produce grains. Yield reduction due to yellow stem borer is estimated to be 1%–19% in early planted and 38%–80% in late transplanted crops (5). The rice leaf folder *C. medinalis* folds the leaves longitudinally and feeds within the green matter, resulting in linear pale white stripe damage. In case of severe infestation, damaged portions are dried up entirely, and the crop gives a whitish appearance. Even a single larva can destroy many leaves by feeding (6). It is also reported that a 10% increase in flag leaf infestation by the leaf folder reduces grain yield by 0.13 g per tiller and the number of filled grains by 4.5% (7).

A wide variety of natural enemies are associated with rice ecosystems. They feed on insect pests as either predators or parasitoids, and this helps to lower the number of insect pests in rice (8). Keeping in mind the significance of natural enemies further, aims should be made to preserve the natural enemies within the rice ecosystem. Some of the important natural enemies in the rice ecosystem are predators like spiders and carabid beetles and egg parasitoids like *Tetrastichus schoenobii* and *Telenomus dignus*, which prey or parasitize on yellow stem borer and leaf folder.

The use of synthetic insecticides is widely adopted by the farming community for the management of these major insect pests. However, in addition to direct toxicity to users, their indiscriminate use has led to environmental pollution, pesticide resistance, pest resurgence, and lethal effects on non-target organisms in agro-ecosystems (9, 10). For this, in recent years, research has focused on the use of botanical pesticides against insect pests (11, 12). Botanical pesticides are very important to the rural poor farmers who are vulnerable and marginalized. In addition, they are easily available in their locality, cheaper, and easy to prepare. Although many studies on insecticidal botanicals have been conducted in the past (13–15), there is little literature available on the bioactivity of various botanicals against stem borer, leaf folder, and natural enemies in rice in real field condition. Keeping this in mind, the present investigation was undertaken.

## Methods

### Experimental site

The experiment was conducted at the Regional Research and Technology Transfer Station (RRTTS), Odisha University of Agriculture and Technology (OUAT), Chiplima, Sambalpur, Odisha, India, at a latitude of 20°21′N and a longitude of 80°55′E with an elevation of 178.8 m above mean sea level (MSL) during the wet seasons of 2022 and 2023. Its climate is warm/sub-humid with an annual average rainfall of 1,426 mm. Its peak rainfall is received during July–August.

### Details of the treatments

Six botanicals were tested for their efficacy in managing lepidopteran pests and natural enemies of rice with one biopesticide and one chemical pesticide (as positive controls) and untreated control. The details of the treatments undertaken in the study are presented in **Table 1**. The experiment was laid out in a randomized complete block design (RCBD) with three replications. The size of each plot measured 5 m × 4 m. Twenty-five-day-old seedlings (Variety MTU 7029, duration 140–145 days) were transplanted in the main field at a spacing of 20 cm × 15 cm (R-R × H-H) during the first week of August for all the two years. At the time of transplanting, three healthy seedlings were taken and transplanted in each hill for all the treatments throughout the field. Weeds were removed from the field manually. Fertilizers and irrigation were applied in the field as per recommended agronomic practices, and no plant protection measures were taken during the crop growth period.

**Table 1:** Details of treatments.

Sl. no.	Treatments	Common name/trade name	Family	Doses used (g or mL/L of water)
T_1_	*Azadirachta indica*, fresh leaves	Neem	Meliaceae	300
T_2_	*Lantana camara*, fresh leaves	*Lantana*	Verbenaceae	300
T_3_	*Nerium oleander*, fresh leaves	Oleander	Apocynaceae	300
T_4_	*Aegle marmelos*, fresh leaves	Bael	Rutaceae	300
T_5_	*Allium sativum*, clove	Garlic	Amaryllidaceae	50
T_6_	*Citrus limon*, fruit	Lemon	Rutaceae	100
T_7_	Azadirachtin 1% EC	Neemazal (EID Parry India Ltd, India)	–	2
T_8_	Acephate 95 SG	Hunk (TATA Rallis, India)	–	1.50
T_9_	Untreated control	–		–

### Preparation of botanical extract

The plant (neem, *Lantana*, *Nerium*, and bael) materials were collected in and around the campus of RRTTS, Chiplima, without cost. After washing and removing water, plant materials were weighed and pulverized using an electrical blender (**Table 1**). Then, pulverized materials were mixed with water with Tween^®^ 20 at 0.1% (Loba Chemie Pvt. Ltd., Mumbai, India) and kept for 24 hrs with frequent stirring just a day before the application date. Tween^®^ 20 was taken due to its non-ionic surfactant properties, and the addition of this to plant extract can improve their efficacy, coverage, stability, and adhesion (9, 16). Thereafter, the solution was filtered using a thin wire mesh followed by a fine cotton cloth. The aqueous extracts were sprayed (**Table 1**) in the field during afternoon hours (after 3 PM) to avoid exposure to direct UV radiation.

### Application of botanical extract

After the preparation of the botanical extract, all the botanicals and chemical insecticides were applied at 20, 35, 50, and 65 days after transplanting (DAT) except untreated control. The knapsack sprayer (ASPEE, Mumbai, India) at 16-L capacity fitted with a hollow cone nozzle was used for a foliar spray of various treatments at the recommended level of 500 L spray volume per hectare. After every treatment, spray nozzles and pipes were washed twice thoroughly with clean water. Every care was taken to minimize drift and contamination of adjacent plots at the time of spraying.

### Methods of recording observations

Major insect pests and natural enemies were identified using available keys and guides from various references such as Dale (2)Barrion and Litsinger (3)Terada and Wu (17), and Mishra et al. (18). Observations were recorded on the incidence of insect pests and natural enemies from 10 randomly selected hills per plot, after each spraying as follows.

Stem borer-infested dead heart (DH) count on 10 plants based on stratified random sampling was recorded at 7 days after each application along with total tillers. The same method was followed for white ear-head infested by stem borer at the time of harvesting along with total productive tillers. The percent incidence of stem borer was calculated as follows:


$$\text{Dead hearts }\left(\text{DH \%}\right)=\frac{\text{No}.\text{of dead heart in }10\text{ hills}}{\text{Total number of tillers in }10\text{ hills}}\;\times 100$$

$$\text{White ear head }\left(\text{WEH \%}\right)=\frac{\text{No}.\text{of white ear heads in }10\text{ hills}}{\text{Total number of productive tillers in }10\text{ hills}}\;\times 100$$

In the case of the leaf folder, the damaged leaves and total leaves from each of the 10 random hills were recorded after 7 days of each application. The percent damage of leaf folder LFDL was calculated as


$$\text{LFDL }\left(\%\right)=\frac{\text{Total number of damaged leaves in }10\text{ hills }\left(\text{one}-\text{third or more of the leaf area damaged}\right)}{\text{Total number of leaves observed in }10\text{ hills}}\;\times 100$$

The reduction percentage of DH and LFDL for each treatment were determined to compare with the untreated control and calculated using the formula


$$\text{Reduction \% over control}=\frac{\text{Mean \% DH or LFDL in control}-\text{Mean \% DH or LFDL for each treatment}}{\text{Mean \% DH or LFDL in control}}\;\times 100$$

Various natural enemies like predators such as spiders and carabid beetles were recorded through visual counting of their motile stages from randomly selected 10 hills 7 days after each spray. To know the natural parasitism of YSB egg masses, the egg masses were collected at 3-day intervals after one spray to the next subsequent spray from the treated and untreated plots and kept in Petri plates with moist filter paper to avoid drying of leaves. Then, the egg masses were observed for the emergence of the adult parasitoids. The emerged adult parasitoids were observed under a stereo-zoom microscope to identify the respective species and numbers. The extent of parasitism of egg masses of YSB was worked out (19).


$$\text{Parasitism }\left(\%\right)=\frac{\text{No}.\text{ of parasitized egg mass}}{\text{No}.\text{ of eggmass collected}}\;\times 100$$

### Grain yield and cost–benefit analysis

The crop was harvested when all grains were matured in the plots. After sun drying (moisture level 15%), the grain yield data (kg/plot) were recorded and converted to ton per hectare. The total income was calculated based on the selling price per quintal of rice. There was no additional profit gained from the botanical-sprayed produce. To find the net profit per hectare for each treatment, the total cost of plant protection was subtracted from the total revenue. The benefit for each sprayed treatment compared to the unsprayed control was calculated by subtracting the income of the control treatment from that of each sprayed treatment. The cost–benefit ratio for each treatment was calculated by subtracting the income of the control treatment from the net income of each sprayed treatment and then dividing the products by the total cost of plant protection for each treatment (13).

### Statistical analysis

The data obtained for various insect pest and natural enemy counts were suitably transformed as suggested by Gomez and Gomez (20) and analyzed using SPSS 16 statistical software (Chicago, IL, USA). The data recorded on grain yield were also subjected to statistical analysis after converting per plot yield to t/ha. Treatment variations were tested for their significance by mean standard error, i.e., SE (m) ± and critical difference (CD) at a 5% level of significance.

## Results

### Effect of different treatments on yellow stem borer (DH and WEH %)

**Figure 1:**
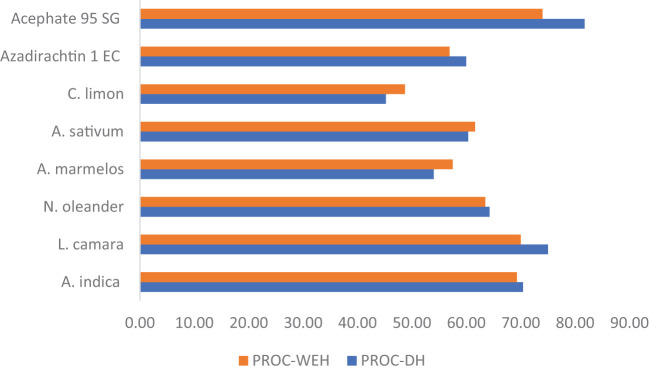
Mean percent reduction of stem borer in terms of dead heart and white ear head (DH and WEH%) against various treatment schedules.

The mean number of dead hearts produced by YSB in various treatment schedules during the wet seasons of 2022 and 2023 (pooled data are shown in **Table 2**) revealed that all the treatments were significantly superior to untreated control. At the early stage among botanicals, after the first spray, neem-, *Lantana*-, *Nerium*-, and garlic-treated plots registered significantly lower percent of dead hearts (0.41%, 0.20%, 0.42%, and 0.40%, respectively) than other treatments and were significantly at par with each other. After the second spray, the efficacy of the neem- and *Lantana*-treated plots registered a significantly lower percentage of dead hearts (1.09% and 0.84%, respectively) than other treatments and was significantly at par with each other. Similarly, after the third and fourth sprays, differences in damage symptoms between various treatments were observed and retained a similar trend of efficacy as found during the second spray. However, among all the treatments, Acephate 95 SG was the most effective (followed by *Lantana*, neem, *Nerium*, garlic, Azadirachtin 1% EC, bael, and lemon). Finally, the overall mean data showed that Acephate 95 SG was the most effective and statistically significant against YSB in terms of dead hearts produced (1.10%) than all other treatments followed by *Lantana* (1.51%), neem (1.78%), *Nerium* (2.16%), garlic (2.39%), Azadirachtin 1% EC (2.42%), bael (2.78%), lemon (3.31%), and untreated control (6.04%). The percent reduction in mean dead heart over control is presented in **Figure 1**. The highest reduction in dead heart percentage (81.78%) was recorded in treatment T_8_ (Acephate 95 SG), followed by T_2_ (*Lantana camara*) (75.02%), T_1_ (*Azadirachta indica*) (70.46), T_3_ (*Nerium oleander*) (64.28%), T_5_ (*Allium sativum*) (60.39%), T_7_ (Azadirachtin 1% EC) (60%), T_4_ (*Aegle marmelos*) (54.02%), and T_6_ (*Citrus limon*) (45.23%).

**Table 2:** Field efficacy of different treatment schedules against stem borer in terms of dead hearts (DH%) and white ear head (WEH%) during wet seasons of 2022 and 2023 (pooled).

Tr. no.	Treatment name	Mean percent dead hearts (DH%)* after 7 days of each spray					WEH%
		4th wk Aug. (27 DAT**)	3rd wk Sep. (42 DAT)	2nd wk Oct. (57 DAT)	1st wk Nov. (72 DAT)	OM	3rd wk Dec. (115 DAT)
1	*Azadirachta indica*	0.41 (0.91)	1.09 (1.25)	2.25 (1.65)	3.39 (1.97)	1.78 (1.51)	3.88 (2.09)
2	*Lantana camara*	0.20 (0.82)	0.84 (1.16)	1.93 (1.56)	3.06 (1.88)	1.51 (1.41)	3.79 (2.07)
3	*Nerium oleander*	0.42 (0.91)	1.39 (1.37)	2.95 (1.86)	3.88 (2.09)	2.16 (1.63)	4.62 (2.26)
4	*Aegle marmelos*	0.82 (1.11)	1.78 (1.51)	3.53 (2.00)	4.97 (2.34)	2.78 (1.81)	5.37 (2.42)
5	*Allium sativum*	0.40 (0.90)	1.67 (1.47)	3.17 (1.92)	4.34 (2.20)	2.39 (1.70)	4.85 (2.31)
6	*Citrus limon*	1.04 (1.19)	2.09 (1.61)	4.17 (2.16)	5.93 (2.54)	3.31 (1.95)	6.49 (2.64)
7	Azadirachtin 1% EC	0.61 (1.02)	1.94 (1.56)	3.18 (1.92)	3.94 (2.10)	2.42 (1.70)	5.44 (2.44)
8	Acephate 95 SG	0.00 (0.71)	0.54 (1.01)	1.38 (1.37)	2.48 (1.72)	1.10 (1.26)	3.28 (1.94)
9	Untreated control	1.88 (1.53)	4.49 (2.23)	8.13 (2.93)	9.68 (3.19)	6.04 (2.56)	12.65 (3.63)
S.Em ±		0.17	0.08	0.07	0.05	0.04	0.05
CD 0.05%		0.50	0.23	0.21	0.16	0.11	0.15

OM, overall mean; SE (m), standard error mean; CD, critical difference.

**DAT, days after transplanting.

*Figures in parentheses are √x + 0.5 transformed values.

Similarly, WEH produced by YSB was found to be reduced significantly in various treatment schedules over untreated control during both the years 2022 and 2023 (**Table 2**). It was observed that the *Lantana* and neem treatments were statistically at par with each other, while Acephate 95 SG was found to be the most effective against yellow stem borer (WEH 3.28%). The percent reduction in mean white ear head over control is presented in **Figure 1**. The highest reduction in white ear head percentage (74.03%) was recorded in treatment T_8_ (Acephate 95 SG) followed by T_2_ (*L. camara*) (70.03%), T_1_ (*A. indica*) (69.32), T_3_ (*N. oleander*) (63.51%), T_5_ (*A. sativum*) (61.64%), T_4_ (*A. marmelos*) (57.54%), T_7_ (Azadirachtin 1% EC) (56.96%), and T_6_ (*C. limon*) (48.73%).

### Effect on leaf folder

**Figure 2:**
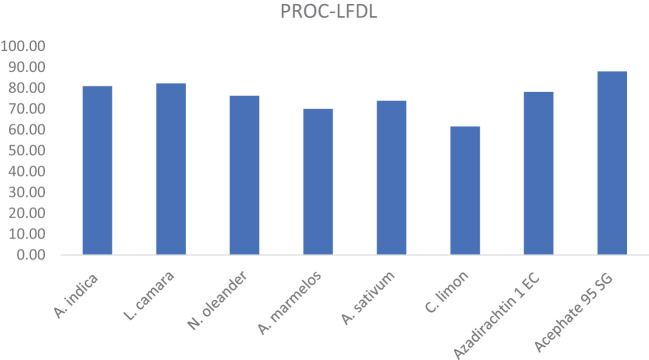
Mean percent reduction of leaf folder damaged leaves (LFDL%) against various treatment schedules.

The mean percent of leaf folder damaged leaves in various treatment schedules during the wet seasons of 2022 and 2023 (pooled data are shown in **Table 3**), which revealed that all the treatments were significantly superior over untreated control. At the initial stage, after the first spray, a low incidence of leaf folder damaged leaves was encountered in all the treated plots. However, their incidence increased after the second spray, which coincided with the third week of September. After the second spray, Acephate 95 SG-, *Lantana*-, and neem-treated plots registered significantly lower incidence of leaf folder damaged leaves (0.69%, 1.18%, and 1.24%, respectively) than other treatments, and the *Lantana* and neem treatments were statistically at par with each other. After the third spray, among botanicals, the *Lantana*-treated plot had a significantly lower incidence of leaf folder damaged leaves (1.25%) but was at par with the neem treatment (1.40%), whereas it was less effective than the Acephate 95 SG-treated plot (0.97%). After the fourth spray, variations in LFDL among different treatments were observed and retained a similar trend of efficacy as found during the third spray. Finally, the overall mean data showed that Acephate 95 SG was the most effective and statistically significant against leaf folder in terms of LFDL (0.43%) than all other treatments, followed by *Lantana* (0.63%), neem (0.68%), Azadirachtin 1% EC (0.78%), *Nerium* (0.84%), garlic (0.93%), bael (1.07%), lemon (1.37%), and untreated control (3.56%). The percent reduction in mean leaf folder damaged leaves over control is presented in **Figure 2**. The highest reduction of leaf folder damaged leaves (87.96%) was recorded in the Acephate 95 SG treatment, followed by the *L. camara* (82.18%), *A. indica* (80.81%), Azadirachtin 1% EC (78.08%), *N. oleander* (76.28%), *A. sativum* (73.81%), (*A. marmelos*) (70%), and *C. limon* (61.53%) treatments.

**Table 3:** Field efficacy of different treatment schedules against leaf folder (LF) in terms of LF damage leaves (LFDL%) during wet seasons of 2022 and 2023 (pooled).

Tr. no.	Treatment name	Mean percent of LFDL* after 7 days of each spray				
		4th wk Aug. (27 DAT**)	3rd wk Sep. (42 DAT)	2nd wk Oct. (57 DAT)	1st wk Nov. (72 DAT)	OM
1	*Azadirachta indica*	0.08 (0.76)	1.24 (1.32)	1.40 (1.38)	0.93 (1.19)	0.68 (1.19)
2	*Lantana camara*	0.07 (0.75)	1.18 (1.29)	1.25 (1.32)	0.88 (1.18)	0.63 (1.16)
3	*Nerium oleander*	0.22 (0.84)	1.50 (1.41)	1.59 (1.44)	1.20 (1.30)	0.84 (1.27)
4	*Aegle marmelos*	0.30 (0.89)	2.00 (1.58)	1.98 (1.57)	1.42 (1.39)	1.07 (1.39)
5	*Allium sativum*	0.15 (0.80)	1.72 (1.49)	1.87 (1.54)	1.23 (1.32)	0.93 (1.32)
6	*Citrus limon*	0.29 (0.89)	2.66 (1.78)	2.68 (1.78)	1.67 (1.47)	1.37 (1.52)
7	Azadirachtin 1% EC	0.07 (0.75)	1.59 (1.44)	1.38 (1.37)	1.13 (1.27)	0.78 (1.24)
8	Acephate 95 SG	0.00 (0.71)	0.69 (1.09)	0.97 (1.21)	0.63 (1.06)	0.43 (1.03)
9	Untreated control	0.79 (1.14)	3.78 (2.06)	7.26 (2.78)	7.15 (2.76)	3.56 (2.29)
S.Em ±		0.05	0.05	0.05	0.05	0.04
CD 0.05%		0.14	0.14	0.16	0.16	0.11

OM, overall mean; SE (m), standard error mean; CD, critical difference.

**DAT, days after transplanting.

*Figures in parentheses are √x + 0.5 transformed values.

### Efficacy of different botanicals against natural enemies

#### Effect of different treatments on the spider population

**Figure 3:**
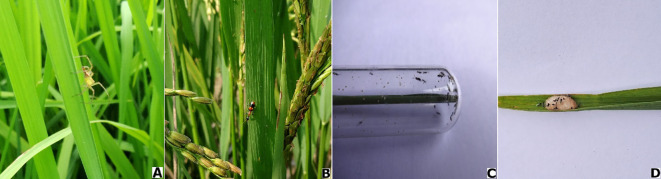
Various predator and parasitoid fauna associated with rice ecosystem at Chiplima, Sambalpur, Odisha: **(A)** lynx spider, *Oxyopes javanus*; **(B)** carabid beetles, *Ophionea indica*; **(C)** *Tetrastichus schoenobii*; **(D)** *Telenomus dignus* and YSB egg mass. YSB, yellow stem borer.

The abundant spider families at Chiplima, Sambalpur, were Araneidae (*Argiope catenulata*), Lycosidae (*Lycosa pseudoannulata*), Tetragnathidae (*Tetragnatha* sp.), Oxyopidae (*Oxyopes javanus*), Linyphiidae (*Erigonidium graminicola*), Theridiidae (*Argyrodes* sp.), and Salticidae (*Plexippus* sp.) (**Table 4** and **Figure 3**). Data on the population of spiders irrespective of species in terms of numbers per 10 hills during the wet seasons of 2022 and 2023 are presented in **Table 4**. After the first spray, no spider populations were observed in different treatments including untreated control. After the second spray, among botanical treatments, the maximum numbers of spiders were observed in bael leaf extract (2.17 per 10 hills), followed by the lemon (1.83 per 10 hills), neem (1.67 per 10 hills), Azadirachtin 1% EC (1.33 per 10 hills), oleander (1.33 per 10 hills), garlic (1.17 per 10 hills), and *Lantana* (1.17 per 10 hills) treatments. No spider population was observed in the Acephate 95 SG-treated plot, but in untreated control, it was 3.00 per 10 hills. Similarly, after the third and fourth sprays, the untreated control plot harbored significantly higher numbers of spider population (9.50 and 19.83 per 10 hills), followed by the bael (7 and 18 per 10 hills), neem (6.50 and 15.67 per 10 hills), Azadirachtin 1% EC (5 and 13.83 per 10 hills), lemon (5 and 13 per 10 hills), *Lantana* (3.83 and 12.17 per 10 hills), garlic (4.33 and 11 per 10 hills), oleander (3.33 and 10.33 per 10 hills), and Acephate 95 SG (0.33 and 2 per 10 hills) treatments.

**Table 4:** Relative effect of different treatment schedules on prevailing spider complex populations in rice ecosystem per 10 hills during wet seasons of 2022 and 2023 (pooled).

Tr. no.	Treatment name	Mean numbers of spider population* after 7 days of each spray			
		3rd wk Sep. (42 DAT**)	2nd wk Oct. (57 DAT)	1st wk Nov. (72 DAT)	OM
1	*Azadirachta indica*	1.67 (1.46)	6.50 (2.64)	15.67 (4.02)	7.94 (2.90)
2	*Lantana camara*	1.17 (1.28)	3.83 (2.08)	12.17 (3.56)	5.72 (2.49)
3	*Nerium oleander*	1.33 (1.35)	3.33 (1.95)	10.33 (3.29)	5.00 (2.34)
4	*Aegle marmelos*	2.17 (1.63)	7.00 (2.73)	18.00 (4.30)	9.06 (3.09)
5	*Allium sativum*	1.17 (1.29)	4.33 (2.20)	11.00 (3.39)	5.50 (2.45)
6	*Citrus limon*	1.83 (1.51)	5.00 (2.34)	13.00 (3.67)	6.61 (2.66)
7	Azadirachtin 1% EC	1.33 (1.35)	5.00 (2.34)	13.83 (3.78)	6.72 (2.69)
8	Acephate 95 SG	0.00 (0.71)	0.33 (0.88)	2.00 (1.58)	0.78 (1.13)
9	Untreated control	3.00 (1.87)	9.50 (3.15)	19.83 (4.51)	10.78 (3.35)
S.Em ±		0.10	0.12	0.08	0.07
CD 0.05%		0.31	0.35	0.24	0.20

OM, overall mean; SE (m), standard error mean; CD, critical difference.

**DAT, days after transplanting.

*Figures in parentheses are √x + 0.5 transformed values.

#### Effect of different treatments on natural parasitism of *S. incertulas* egg mass

A significant number of natural parasitoids of *S. incertulas* egg mass were observed in the experimental field of rice during the wet seasons of 2022 and 2023 (**Tables 5**, **6**; **Figure 3**). At the initial stage of the plant growth period, natural parasitism of *S. incertulas* egg mass was not observed in the rice field, but after 42 DAT coincided with the second spray, they were noticed but were less in number (0%–6.11% in the third to fourth weeks of September). After that, YSB egg mass parasitism rates increased steadily, which were mainly observed after the third and fourth sprays coincided with October (8.33%–52.78%) and November (13.89%–65.28%). The untreated control plot had (overall mean of 41.39% natural parasitism of YSB egg mass) the significantly highest percentage of natural parasitism of YSB egg mass, followed by bael- (overall mean of 36.57%) and neem-treated (overall mean of 33.33%) plots, while they were statistically at par to each other. Among the various treatments, the Acephate 95 SG-treated plot exhibited the lowest percentage of YSB natural parasitism (overall mean 7.41%).

**Table 5:** Relative effect of different treatment schedules on natural parasitism of *Scirpophaga incertulas* egg mass in rice ecosystem during wet seasons of 2022 and 2023 (pooled).

Tr. no.	Treatment name	Mean of natural parasitism of *S. incertulas* egg mass %* after each spray			
		3rd to 4th wk Sep.	2nd to 3rd wk Oct.	1st to 2nd wk Nov.	OM
1	*Azadirachta indica*	5.56 (10.86)	38.89 (38.66)	55.56 (48.54)	33.33 (35.46)
2	*Lantana camara*	0.00 (4.05)	27.78 (31.87)	43.06 (41.28)	23.61 (29.40)
3	*Nerium oleander*	0.00 (4.05)	29.17 (32.70)	44.44 (42.05)	24.54 (30.01)
4	*Aegle marmelos*	6.67 (13.96)	43.33 (41.37)	59.72 (50.95)	36.57 (37.50)
5	*Allium sativum*	3.33 (9.01)	37.50 (38.04)	40.00 (39.47)	26.94 (31.52)
6	*Citrus limon*	4.17 (9.75)	36.11 (37.06)	41.67 (40.48)	27.31 (31.81)
7	Azadirachtin 1% EC	2.78 (8.47)	30.56 (33.14)	36.11 (37.16)	23.15 (28.81)
8	Acephate 95 SG	0.00 (4.05)	8.33 (12.81)	13.89 (19.62)	7.41 (14.55)
9	Untreated control	6.11 (13.42)	52.78 (46.94)	65.28 (54.30)	41.39 (40.30)
S.Em ±		4.34	5.37	3.62	2.54
CD 0.05%		13.00	16.08	10.86	7.62

OM, overall mean; SE (m), standard error mean; CD, critical difference.

*Figures in parentheses are arcsine-transformed values.

**Table 6:** Relative parasitism of YSB egg masses by parasitoid alone or in combination.

Sl. no.	Month	Parasitoid			
		2022	% emergence	2023	% emergence
1	_4th wk Aug._	0	–	0	–
2	_3rd to 4th wk Sep._	0	*-*	*Tetrastichus schoenobii*	62.50%
				*Telenomus dignus*	37.50%
3	_2nd to 3rd wk Oct._	*T. schoenobii*	42.86%	*T. schoenobii*	47.22%
		*T. dignus*	33.33%	*T. dignus*	36.11%
		*T. schoenobii* and *T. dignus*	14.28%	*T. schoenobii* and *T. dignus*	11.11%
		*Trichogramma japonicum*	9.52%	*T. japonicum*	5.55%
4	_1st to 2nd wk Nov._	*T. schoenobii*	41.67%	*T. schoenobii*	38%
		*T. dignus*	36.11%	*T. dignus*	44%
		*T. schoenobii* and *T. dignus*	13.89%	*T. schoenobii* and *T. dignus*	14%
		*T. japonicum*	8.33%	*T. japonicum*	4%

YSB, yellow stem borer.

From the YSB egg mass, three parasitoids, namely, *T. schoenobii*, *T. dignus*, and *Trichogramma japonicum*, emerged. The relative parasitism of YSB egg masses by various parasitoids revealed that initially (September–October), *T. schoenobii* was dominant, but later, *T. dignus* also played a significant role in YSB egg mass parasitism (November). The activity of *T. japonicum* was maximum in November (6.16%). The relative parasitism of YSB egg mass by different species of egg parasitoid revealed a mean of 12.69% and 13.94% parasitism by two species (*T. schoenobii* and *T. dignus*) during the last week of October and the first week of November. In addition, no parasitized egg mass was observed, which was attacked by more than two egg parasitoids.

#### Effect of different treatments on carabid beetles

The carabid beetles *Ophionea indica* (Thunberg) are the predators of leaf folders and also prey on plant hoppers. Each predator may consume three to five leaf folder larvae per day, leaving only the head capsule (21). The adult population of carabid beetles *O. indica* (Thunberg) varied significantly among various treatments during the wet seasons of 2022 and 2023 (**Table 7**; **Figure 3**). The Acephate 95 SG-treated plot (0.25 per 10 hills) exhibited the lowest number of populations followed by the oleander leaf extract-treated plot, while the untreated plot (2.33 per 10 hills) and bael- (2.00 per 10 hills) and neem-treated (1.67 per 10 hills) plots had more numbers of carabid beetles.

**Table 7:** Relative effect of different treatment schedules on prevailing carabid beetles (*Ophionea indica*) populations in rice ecosystem per 10 hills during wet seasons of 2022 and 2023 (pooled).

Tr. no.	Treatment name	Mean numbers of carabid beetles* after 7 days of each spray		
		2nd wk Oct. (57 DAT**)	1st wk Nov. (72 DAT)	OM
1	*Azadirachta indica*	1.33 (1.33)	2.00 (1.58)	1.67 (1.47)
2	*Lantana camara*	1.00 (1.21)	1.50 (1.41)	1.25 (1.32)
3	*Nerium oleander*	0.67 (1.05)	1.00 (1.22)	0.83 (1.15)
4	*Aegle marmelos*	1.83 (1.53)	2.17 (1.63)	2.00 (1.58)
5	*Allium sativum*	0.83 (1.14)	1.50 (1.41)	1.17 (1.29)
6	*Citrus limon*	1.50 (1.41)	2.00 (1.58)	1.75 (1.50)
7	Azadirachtin 1% EC	1.00 (1.21)	1.17 (1.29)	1.08 (1.25)
8	Acephate 95 SG	0.17 (0.80)	0.33 (0.88)	0.25 (0.84)
9	Untreated control	2.17 (1.63)	2.50 (1.73)	2.33 (1.68)
S.Em ±		0.12	0.10	0.08
CD 0.05%		0.35	0.30	0.24

OM, overall mean; SE (m), standard error mean; CD, critical difference.

**DAT, days after transplanting.

*Figures in parentheses are √x + 0.5 transformed values.

### Yield and economics

Mean healthy seed yields of rice were significantly higher in all the treatments over the untreated control (**Table 8**), and significant differences were observed among the treatments. Based on yield, the Acephate 95 SG-treated plot recorded the highest mean grain yield of 4.68 t/ha and was found statistically at par with the neem-treated plot (4.66 t/ha). The highest yield increase over control was in the insecticidal treatment Acephate 95 SG (51.44% over control), followed by the neem- (51.27% over control) and bael-treated plots (49.46% over control). The neem treatment had the best cost–benefit ratio of 1:3.74. Following closely was the bael treatment, which had a cost–benefit ratio of 1:3.41. Plots sprayed with Acephate demonstrated a cost–benefit ratio of 1:4.65. The lowest cost–benefit ratio of 1:1.57 was achieved for plots treated with lemon fruit.

**Table 8:** Yield of rice grains (t/ha) and economics of different treatment schedules employed to manage lepidopteran insect pests during wet seasons of 2022 and 2023.

Tr. no.	Yield (t/ha)		Mean yield (t/ha)	Yield increase over control (%)	Extra expenditure* (₹/ha) for treatment over control	Net income** (₹/ha) over control	Cost–benefit ratio (C:B)
	2022	2023					
1	4.73 (2.29)	4.58 (2.25)	4.66 (2.27)	51.27	11,000	41,137	1:3.74
2	4.42 (2.22)	4.38 (2.21)	4.40 (2.21)	48.41	11,000	35,498	1:3.23
3	4.37 (2.21)	4.33 (2.20)	4.35 (2.20)	47.82	11,000	34,406	1:3.13
4	4.55 (2.25)	4.43 (2.22)	4.49 (2.23)	49.46	11,000	37,499	1:3.41
5	4.48 (2.23)	4.40 (2.21)	4.44 (2.22)	48.89	13,900	33,507	1:2.41
6	4.32 (2.19)	4.20 (2.17)	4.26 (2.18)	46.69	16,900	26,505	1:1.57
7	4.35 (2.20)	4.32 (2.19)	4.33 (2.20)	47.62	10,200	34,843	1:3.42
8	4.82 (2.31)	4.53 (2.24)	4.68 (2.27)	51.44	9,300	43,201	1:4.65
9	2.35 (1.69)	2.18 (1.64)	2.27 (1.66)	–	–	–	–
S.Em ±	0.01	0.02	0.01				
CD 0.05%	0.04	0.05	0.04				

SE (m), standard error mean; CD, critical difference.

*Average laborer charge at ₹ 350/day; neem, *Lantana*, *Nerium* and bael plant parts obtained without cost; Tween 20 at ₹ 1,000/500 mL; Acephate 95 SG at ₹ 1,700/kg; garlic at ₹ 200/kg; lemon at ₹ 15/100 g; Neemazal 1 EC at ₹ 1,500/L.

**Rice minimum support price as per Govt. of India in 2023 (MSP) ₹ 2,183/q.

## Discussion

From the results, it was observed that all the botanicals tested in this study were effective in minimizing the infestation of major lepidopteran insect pests of rice as compared to the untreated control. Among various botanicals, A*. indica* extract was highly effective against both the lepidopteran insects, whereas *L. camara* extract was the most promising against YSB. The result found in this investigation is somewhat in conformity with the findings of Ogah et al. (22), who reported that neem seed kernel extract was effective against rice insect pests. In another experiment, Parajuli et al. (23) found that crude water extracts of green neem leaves at 200 g of leaves per liter of water were effective against cabbage butterfly (*Pieris brassicae* Lin.), soybean hairy caterpillar (*Spilosoma obliqua* Walker), and tobacco caterpillar (*Spodoptera litura* Fab.). Gonzales (24) found that *L. camara* had insecticidal properties against rice insect pests, especially stem borer.

The result also revealed that all the botanical extracts were safe from natural enemies in comparison to synthetic chemicals. It is also observed that among botanicals, bael- and neem extract-treated plots had very little impact on spider fauna, egg parasitoids of stem borer, and carabid beetles. The natural parasitism of egg masses of YSB was minimal in August, whereas it was maximum in November. *T. schoenobii*, *T. dignus*, and *T. japonicum* were the three parasitoids observed in the egg masses collected from the experimental plots. Among them, *T. schoenobii* was the most prevalent, followed by *T. dignus* and *T. japonicum*. Our result is in conformity with the findings of Manjunath (25), who reported maximum parasitization of YSB egg masses by *Tetrastichus* sp., followed by *Telenomus* sp. Similarly, our results conformed with the findings of Williams and Mansingh (26), who reported that the application of neem extracts to a rice field did not hamper the activity of *L. pseudoannulata* Boes and Strand (Araneae: Lycosidae).

It was observed that although the numbers of many major insect pests’ on crops treated with pesticidal plants were significantly higher than observed with the synthetic control, crop yields obtained from pesticidal plant treatments were comparable to those from the synthetic pesticide treatment. This is most notable with the use of neem leaf extract (4.66 t/ha), where the yields were statistically at par with those of synthetic insecticide (4.68 t/ha). Bael leaf also performed well (4.49 t/ha) in terms of rice production. This could be due to further pest reduction in botanical extract through natural enemies, as more numbers of natural enemies were present in those treatments. Similar types of findings were observed by various workers who reported that botanicals were less harmful to natural enemies than synthetic insecticides (9, 27, 28). Although *Lantana* leaf extract was effective against both the lepidopteran pests, production was low in comparison to that of neem extract, which may be due to the other pest infestation in the treated plot. The insecticide Acephate 95 SG significantly reduced the yellow stem borer and leaf folder infestation compared to the untreated control.

The cost–benefit ratio serves as an indicator of the relative economic performance of the treatments. An indicator above 1 demonstrates the economic viability of the treatment when compared to the control treatment. Within this study, cost–benefit ratios ranging from 1:1.57 to 1:4.65 signify that the treatments were biologically effective and yielded a significant return on investment in plant protection. Among botanicals, neem and bael proved to be more economically viable, while the synthetic chemical Acephate was marginally superior compared to all other treatments. Since all botanicals provided cost–benefit ratios exceeding 1, growers have the flexibility to choose from a variety of botanicals for beneficial spray extracts. The cost–benefit ratios calculated in this study are quite similar to those reported by Shabozoi et al. (29) but are lower than the ratios obtained by Amoabeng et al. (13). Shabozoi et al. (29) achieved a cost–benefit ratio of 1:4.1 when using a neem-based botanical to control insect pests in okra, while Amoabeng et al. (13) achieved ratios of 1:29 and 1:18 for botanical (*Chromolaena odorata* extract) and synthetic insecticide (Emamectin benzoate), respectively, in managing insect pests of cabbage. In addition, the price of the product and treatment significantly impacts the cost–benefit ratio, overall income, and the benefit derived from each treatment. According to the findings of this research, although the garlic treatments resulted in a moderate yield, the total income and cost–benefit ratio were adversely affected by the high market price.

Synthetic insecticides are often misused, leading to negative impacts on ecosystems and human health, particularly in developing countries. Using botanicals such as extracts of pesticidal plants has long been argued to be more sustainable and appropriate for smallholder farmers in developing countries (11, 30), and here, it was also observed that the use of pesticidal plants can effectively manage insect pests and can be integrated into sustainable agricultural practice. The findings of the present study therefore indicate that leaves of studied botanicals have some toxic properties against insect pests. Various workers also suggested that several bioactive components in botanical extract were responsible for their insecticidal action. For example, garlic has insecticidal activity due to the presence of the major bioactive components like diallyl sulfide, diallyl disulfide, diallyl tetrasulfide, dimethyl trisulfide, and 3-vinyl-[4*H*]-1,2-dithiin (14). Similarly, the major bioactive components of *L. camara* are germacrene D, *b*-caryophyllene, *a*-phellandrene, limonene, and 1,8-cineole (31). Bioactive constituents from leaves of *N. oleander* include oleandrin, oleandrigenin, digoxin, digitonin, digitoxigenin, nerizoside, neritaloside, and odoroside (32). Chemical analysis of neem has indicated the presence of various active ingredients like azadirachtin, salannin, meliantriol, and nimbin, which contain insecticidal and pesticidal properties (33–35). Leaves of *A. marmelos* contain alkaloids, mermesinin, rutin, phenylethyl cinnamides, anhydromarmeline, and aegelinosides, which have insecticidal and antifeedant properties (36–38). Bioactive constituents from *C. limon* include limonene, farnesol, and palatinol-1C (39).

This research has demonstrated that inexpensive plant extracts from commonly found plants can serve as affordable alternatives to synthetic insecticides for protecting plants. However, the active compounds in plant extracts break down quickly and have limited persistence (40), which require more applications than synthetic pesticides; hence, labor inputs may rise when utilizing crude preparations of pesticidal plants. However, the lower persistence of pesticidal plants means that the health of the applicator and consumers is less in danger owing to reduced exposure to bioactive compounds from the plants, which turn into harmless natural products. This implies that there is no need to worry about harmful residues in food grains when harvesting crops. It also makes it possible for growers to produce for export and higher-end organic markets. Thus, all the tested plants showed promising results against rice insect pest management, but among those, aqueous extracts of neem, *Lantana*, and bael leaves were most effective in terms of production as well as suppression of major insect pests in rice and did not affect the beneficial insects. Therefore, they may be included in integrated pest management in rice.

## Data Availability

The datasets presented in this study can be found in online repositories. The names of the repository/repositories and accession number(s) can be found in the article/supplementary material.
